# Activation of the PERK/MANF/STAT3 Pathway in Astrocytes Promotes Synaptic Remodeling and Neurological Recovery in the Acute Phase After Stroke in Mice

**DOI:** 10.1155/np/6776608

**Published:** 2025-08-28

**Authors:** Yashu Sun, Lan Luo, XiaoYan Li, Bing Zhang

**Affiliations:** Department of Anesthesiology, The Second Affiliated Hospital of Harbin Medical University, Harbin, Heilongjiang, China

**Keywords:** astrocyte, inflammation, MANF, PERK, STAT3, stroke, synaptic remodeling

## Abstract

Astrocytes play a crucial role in ensuring neuronal survival and function. In stroke, astrocytes trigger the unfolded protein response (UPR) to restore endoplasmic reticulum homeostasis. Mesencephalic astrocyte-derived neurotrophic factor (MANF), a newly identified endoplasmic reticulum stress-induced neurotrophic factor, attenuates cerebral ischemic injury by reducing inflammatory responses. The mechanisms by which astrocytes regulate MANF expression and the role of MANF in modulating inflammation remain to be elucidated. In this study, we constructed middle cerebral artery occlusion (MCAO)/reperfusion model in C57BL/6J mice and an oxygen glucose deprivation/reoxygenation model in a neuronal and astrocyte coculture system. The present study utilized an intraventricular injection of adeno-associated virus (AAV) to effectively block the PERK pathway in astrocytes. Moreover, MANF-siRNA was employed to suppress endogenous MANF expression, while rhMANF was used as an exogenous supplement. 2,3,5-Triphenyltetrazolium chloride (TTC), modified neurological severity score (mNSS), adhesive removal test, Golgi staining, hematoxylin-eosin (HE) staining, western blot, and enzyme-linked immunosorbent assay (ELISA) were applied to evaluate the protective effects of PERK pathway and the expression of MANF in astrocytes. In vitro experiments, ELISA, cell counting kit-8 (CCK-8), and western blot were used to detect the mechanisms by which MANF regulates neuroinflammation. The results showed that blocking the astrocytic PERK pathway decreased MANF expression, aggravated synaptic loss, and exacerbated infarct volume and neurological outcomes. Conversely, cellular experiments showed that activation of PERK increased MANF expression, promoted synaptic protein expression, and increased neuronal cell viability. Additionally, increasing exogenous MANF inhibited STAT3 phosphorylation, reduced the release of inflammatory factors, and improved neuronal cell viability. In conclusion, our study demonstrates that after stroke, astrocytes activate PERK and upregulate MANF expression, which inhibits STAT3 phosphorylation, reduces proinflammatory cytokine release, rescues neuronal synapse loss, and promotes the recovery of neurological function in mice.

## 1. Introduction

Ischemic stroke is one of the leading causes of death and disability worldwide [1]. Ischemic stroke causes a variety of pathological changes, including neuronal death, brain edema, and synaptic damage. The integrity of neuronal and synaptic networks is disrupted after stroke, leading to impaired sensory, motor, and cognitive function [2]. However, the neurological system has a regenerative and repair potential that can promote the recovery of neurological function after stroke through synaptic plasticity, neuronal regeneration, increased interhemispheric connection, and other mechanisms [3, 4]. Among them, synaptic plasticity is an important factor in the recovery of neurological function after a stroke.

When cells are ischemic and hypoxic, misfolded proteins accumulate in large quantities in the endoplasmic reticulum and activate the unfolded protein response (UPR) [5]. UPR is a protective mechanism by which cells respond to endoplasmic reticulum stress and is closely associated with brain injury [6, 7]. When cerebral ischemia-reperfusion occurs, large amounts of reactive oxygen species are generated, which interfere with protein folding, processing, and degradation processes and further activate the UPR [8, 9]. The UPR is mediated by three classical transmembrane sensors of endoplasmic reticulum stress, including PERK, IRE1, and ATF6, which activate a series of downstream signaling molecules that regulate the expression of certain genes and restore endoplasmic reticulum homeostasis. Unlike the IRE1 and ATF6 branches, which mainly play a protective role after stroke, the PERK pathway can either restores endoplasmic reticulum homeostasis or activates cell death processes [10]. Activation of PERK promotes phosphorylation of eukaryotic translation initiation factor 2α (eIF2α), which further activates the translation of transcription factor 4 (ATF4), thus, upregulating the translation level of UPR target genes to enhance the endoplasmic reticulum's protein folding, antioxidant response, and autophagy. However, most of the above studies are focused on neurons, and the effects of astrocytes under UPR stress on neurological function after stroke and the related mechanism are still unclear.

Astrocytes play multiple important roles in the central nervous system, typically responsible for maintaining the stability of the brain environment and providing nourishment and support to neurons [11]. After stroke, activated astrocytes play an important role in disease progression [12]. Mesencephalic astrocyte-derived neurotrophic factor (MANF) is a member of the neurotrophic factor family. In intact brain, MANF is predominantly expressed in neurons, while after stroke, MANF expression is downregulated in neurons and upregulated in microglia and astrocytes [13]. In stroke models, MANF has been shown to protect neural stem cells from hypoxic and glycolytic injury [14]. Injection of MANF into the lateral ventricle promoted neurological recovery, brain remodeling around the lesion, and lesion-remote axon plasticity in mice after ischemia [15]. In addition, MANF alleviated alcoholic liver injury by activating STAT3-mediated autophagy [16]. However, the mechanism underlying the upregulation of MANF expression in astrocytes after stroke is not clear, and its mechanism of promotion of neurological recovery needs to be further explored. In our study, we hypothesized that after stroke astrocytes activate the PERK-elf2α pathway, upregulate MANF, and inhibit STAT3 phosphorylation, thereby, attenuating the release of inflammatory factors, promoting synaptic plasticity in the ischemic penumbra regions, and ameliorating ischemia-reperfusion injury.

## 2. Materials and Methods

### 2.1. Animals and Middle Cerebral Artery Occlusion (MCAO)

Male C57BL/6J mice of weighting 18–22 g and aged 6–8 weeks old were acquired from Beijing Viton Lever Technology Co., and randomly divided into four groups: Sham group, MCAO group, MCAO + adeno-associated virus (AAV)-gfa-empty, and MCAO + AAV-gfa-GADD34. And the environmental conditions were maintained at a temperature of 25°C, relative humidity of 50%, and a 12-h light–dark cycle. All manipulations followed the principles outlined in the National Institutes of Health's Guide for the Care and Use of Laboratory Animals, and were approved by the Ethics Committee of the Second Affiliated Hospital of Harbin Medical University (SYDW2023-133). The middle cerebral artery (MCA) of mice was blocked by 6–0 nylon monofilament with a round tip under 2% sevoflurane anesthesia, as described previously [17]. The monofilament entered the external carotid artery (ECA), slid into the internal carotid artery (ICA), advanced about 11 mm and ultimately blocked the MCA. After 60 min of MCAO, the nylon monofilament was withdrawn, the ECA was ligated, and the skin was sutured. According to experimental flowchart, mice were euthanized by cervical dislocation on the 1^st^ and 7^th^ day after reperfusion.

### 2.2. Intraventricular Injection

In order to modulate PERK/eIF2a signaling specifically in astrocytes, we generated AAV that overexpress an active fragment of GADD34 (DhuGADD34), the specific inducible phosphatase of P-elf2a, under the astrocytic promoter gfaABC1D. AAV was sourced from WZ Biosciences (Jinan, China). Three weeks before MCAO, mice were anesthetized and placed on a stereotaxic apparatus. According to the “Stereotaxic Atlas of the Mouse Brain,” we designed bregma as the zero point and localized the ventricle as 0.3 mm posterior to bregma, 1 mm away from the center line, and a needle depth of 2.3 mm. Then, 2 μL of AAV-gfa-empty or AAV-gfa-GADD34 virus were slowly injected at this site (AP: −0.3 mm, ML: ±1 mm, and DV: −2.3 mm) using a Hamilton syringe.

### 2.3. Modified Neurological Severity Score (mNSS)

The mNSS was performed on days 1, 3, and 7 after MCAO, as previously described [18]. The total score is 18, and the higher the score, the more severe the neurological damage [19]. The following evaluation criteria are to be employed: balance function tests (0–6), including balance beam walking test; motor function tests (0–6), including tail-hanging tests (0–3) and straight-line walking experiments (0–3); sensory function tests (0–2), including tactile stimulus response and proprioceptive tests; reflex tests (0–4), including pinna reflex, corneal reflex, and startle reflex.

### 2.4. Adhesive Removal Test

The adhesive removal test was used to evaluate the sensorimotor dysfunction and motor asymmetry after stroke [20]. Mice were trained for 3 days before surgery. A piece of tape (0.3 × 0.4 cm^2^) was applied to the right paw of each mouse. The time taken by the mice to remove the tape from paw was recorded on days 1, 3, and 7 after MCAO.

### 2.5. 2,3,5-Triphenyltetrazolium chloride (TTC) Staining

After 24 h of reperfusion, the mice were euthanized, and the brains were frozen and coronal sections were performed. Then, the sections were soaked in 2% TTC (#17779, Sigma) at 37°C for 1 h, fixed in formaldehyde and photographed [21]. The infarct area was calculated using ImageJ software (NIH, Bethesda, USA) and expressed as a percentage of the contralateral hemisphere area.

### 2.6. Golgi Staining and Sholl Analysis

After anesthesia, the mice hearts were perfused with precooled saline, and brain tissues were taken and cultured in Golgi solutions (equal solutions A and B mixtures of potassium dichromate, mercuric chloride, and potassium chromate) for 14 days after anesthesia [22]. The brains were then soaked in 80% glacial acetic acid overnight, washed and placed in 30% sucrose until precipitation, and then sliced to a thickness of 120 μm. Sholl analysis in ImageJ was used to analyze neuronal morphology and dendritic branches. Three fields of view were taken from each brain slice and three neurons were counted in each field of view. Dendritic spine density is the number of dendritic spines per 10 μm at the top of the secondary dendritic branches.

### 2.7. Hematoxylin-Eosin (HE) Staining

After 7 days of reperfusion, the brain tissues were embedded in paraffin, sliced into 4 μm thick sections, and stained with hematoxylin for 5 min. Then, the sections were differentiated in 1% hydrochloric acid alcohol for 10 s, stained with eosin for 5 min, and finally dehydrated twice in ethanol. Seal the sections with neutral resin and observe them under a microscope [23].

### 2.8. Western Blot

Peri-infarct tissue was homogenized in ice-cold RIRP buffer, protease inhibitors, and phosphatase inhibitors. The protein concentrations were determined by BCA kits, and the proteins were boiled and frozen at −80°C. Subsequently, the proteins were separated by sodium dodecyl sulfate-polyacrylamide gel electrophoresis and transferred to 0.45 μm PVDF membranes. The membrane was blocked with 5% skimmed milk and incubated with primary antibodies overnight at 4°C [24]. Primary antibodies included anti-PERK (1:1000, #ab229912, Abcam, Cambridge, MA, USA), anti-phosphorylated PERK (1:1000, #AF4499, OH, USA), anti-elf2α (1:1000, #ab242148), anti-phosphorylated elf2α (1:1000, #ab32157), anti-PSD95 (1:1000, #AF5283), anti-Synaptophysin (1:1000, #5461, Cell Signaling Technology, Danvers, MA, USA), anti-MANF (1:1000, #ab316935), anti-phosphorylated STAT3-Tyr705 (1:1000, #AF3293), and anti-β-actin (1:5000, #bs-10966R, Bioss, Beijing, China). Then, the membranes were then incubated with horseradish peroxidase-conjugated secondary antibody (1:5000, #ZB-2301, ZSGB-Bio, Beijing, China) for 1 h at room temperature, and then the membranes were incubated with ECL chemiluminescence kit (#P10200, NCM Biotech, Suzhou, China) and detected by using a chemiluminescence imaging system (Clinx Science Instruments, China). Protein expression was measured with ImageJ software (1.8.0) and normalized to β-actin.

### 2.9. Cell Culture and Oxygen-Glucose Deprivation (OGD)

C8D1A astrocytes and HT22 neurons were purchased from Procell Life Science & Technology Company (Wuhan, China). The cells were planted in DMEM containing 10% fetal bovine serum and 1%P/S at 37°C in 5% CO_2_ and 95% air. In the following experiments, the cells were used for sub-cultivating, plate laying, preparation of models, and cell coculture. OGD treatment was conducted as described previously [25]. In brief, the medium of the cells was replaced with sugar-free DMEM medium, and then the cells were placed in an incubator containing 95% nitrogen and 5% carbon dioxide. After 4 h, the medium was replaced by DMEM. Finally, the cells were transferred to a humidified incubator at 37°C for 24 h.

### 2.10. Neuron-Astrocyte Coculture System

Neurons were planted in the lower layer of transwell compartment, while astrocytes were planted in the upper layer. OGD was performed after cells adhesion and the astrocytes were pretreated with CCT020301, GSK2656157 or rhMANF. The culture medium and cells from the two layers were collected separately for subsequent experiments.

### 2.11. Transfection of MANF- siRNA

C8D1A cells were evenly spread in a six-well plate at 200,000 cells per well. After the cells adhered to the wall and reached a density of 50%–70%, replaced the culture medium with serum-free culture medium, and 50 nM MANF-siRNA or siNC combined with lip2000 were added to the medium. After 6 h, the medium was replaced with antibiotic-free medium. At the same time, neurons were planted in the lower layer of transwell chamber, to establish a coculture system. After 18 h, astrocytes were treated with OGD or CCT020312.

### 2.12. Drug Pretreatment of Astrocytes

CCT020312, GSK2656157, and rhMANF were purchased from MedChemExpress (Monmouth Junction, NJ, USA). CCT020312 is a PERK signaling pathway activator. The cells were cultivated with final concentration of 5 μM CCT020312. CCT020312 activates the PERK signaling pathway, whereas GSK2656157 inhibits it. Cells were incubated with 5 μM CCT020312 or 5 μM GSK2656157 for 24 h, and then the medium was changed to sugar-free medium. Configure rhMANF to a final concentration of 100 ng/mL and pretreat astrocytes for 1 h before OGD.

### 2.13. Cell Counting Kit-8 (CCK8)

The viability of HT22 cells was evaluated using the CCK-8 assay kit (Cat#C6005, NCM Biotech, Suzhou, China). Neurons were panted at a density of 3000 cells per well. When the cells adhered to the wall, 10 μL of CCK8 reagent was added to each well under dark conditions. After 30 min, the absorbance was measured at 450 nm using a microplate reader [26].

### 2.14. Enzyme-Linked Immunosorbent Assay (ELISA) IL-10, TNF-α, IL-1β

The levels of IL-10, TNF-α, and IL-1β in brain tissue and astrocytes were quantified using ELISA kits according to the manufacturer's instructions (Jingmei Biotech, Nanjing, China).

### 2.15. Data Analysis

Data are presented as mean ± standard deviation (SD) or median (interquartile range) and analyzed using ImageJ and GraphPad Prism 10.0. The Shapiro–Wilk normality test was used to check the normality of continuous data. For multiple comparisons, if the data followed a normal distribution and had homogeneity of variance, one-way analysis of variance (ANOVA) followed by Bonferroni's test was applied. The Kruskal–Wallis test was employed to analyze data with a non-normal distribution. A *p*-value less than 0.05 was considered statistically significant.

## 3. Results

### 3.1. Blocking PERK Phosphorylation in Astrocytes Exacerbated the Decline of Synaptic Proteins in Peri-Infarct Zone, Increased Infarct Size, and Aggravated Neurological Impairment in Mice After Stroke

**Figure 1:**
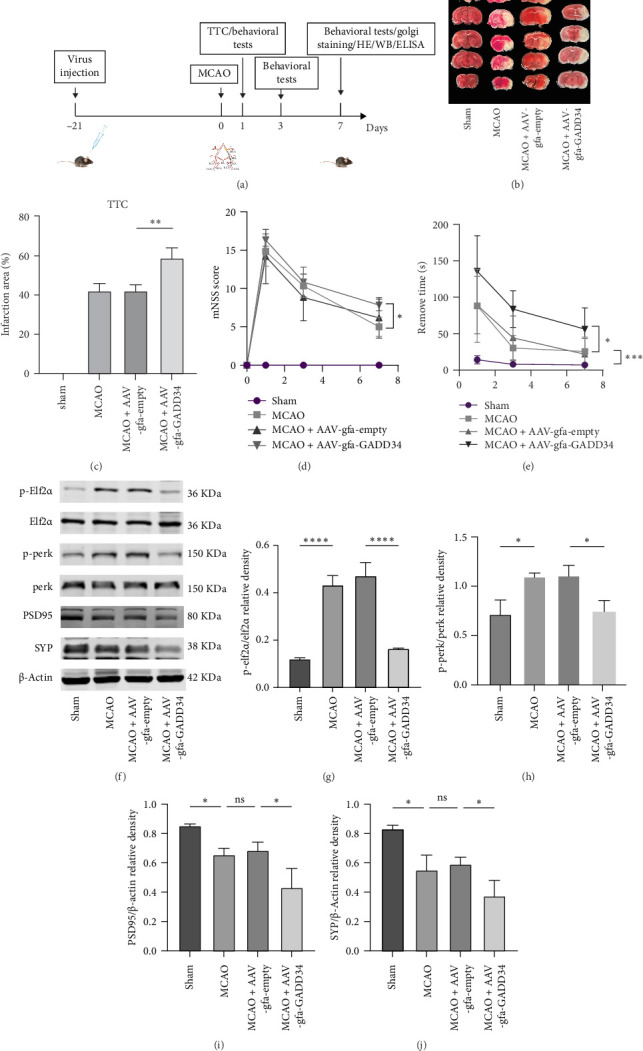
Blocking PERK phosphorylation in astrocytes exacerbated the decline of synaptic proteins in peri-infarct zone, increased infarct size, and aggravated neurological impairment in mice after stroke. (A) Timeline of the experiment. (B, C) Five brain slice images of TTC from one representative mouse from each group and quantitative analysis of infarct volume (*n* = 3). (D) mNSS score (*n* = 6). (E) Adhesive removal test (*n* = 6). (F) Representative images of western blot on the 7^th^ day after MCAO. (G–J) Quantitative analysis of western blot (*n* = 3). ns, Not significant. *⁣*^*∗*^*p* < 0.05, *⁣*^*∗∗*^*p* < 0.01, *⁣*^*∗∗∗*^*p* < 0.001, *⁣*^*∗∗∗∗*^*p* < 0.0001.

To specifically modulate PERK-eIF2α signaling pathway in astrocytes, we generated AAV overexpressing GADD34, the specific inducible phosphatase of eIF2a-P, and placed downstream of the astrocyte-specific promoter gfaABC1D. Mice were injected with the virus before the experiment to allow for viral expression (Figure 1A). As shown in Figure 1, mice in the MCAO + AAV-gfa-GADD34 group had increased infarct size, higher mNSS scores, and prolonged sticker removal time compared with the MCAO group. Compared with the Sham group, the MCAO group activated the phosphorylation of PERK and elf2α, while the MCAO + AAV-gfa-GADD34 group reduced the expression of phosphorylated PERK and elf2α (Figure 1). The expression of the synaptic proteins PSD95 and SYP was reduced in the peri-infarct tissues compared with the Sham group and further reduced in the MCAO + AAV-gfa-GADD34 group (Figure 1I,J).

### 3.2. Blockade of PERK Signaling Pathway in Mice Astrocytes Reduced MANF Expression, Promoted the Release of Inflammatory Factors, and Exacerbated Synapse Loss and Neuronal Damage in the Peri-Infarct Zone

**Figure 2:**
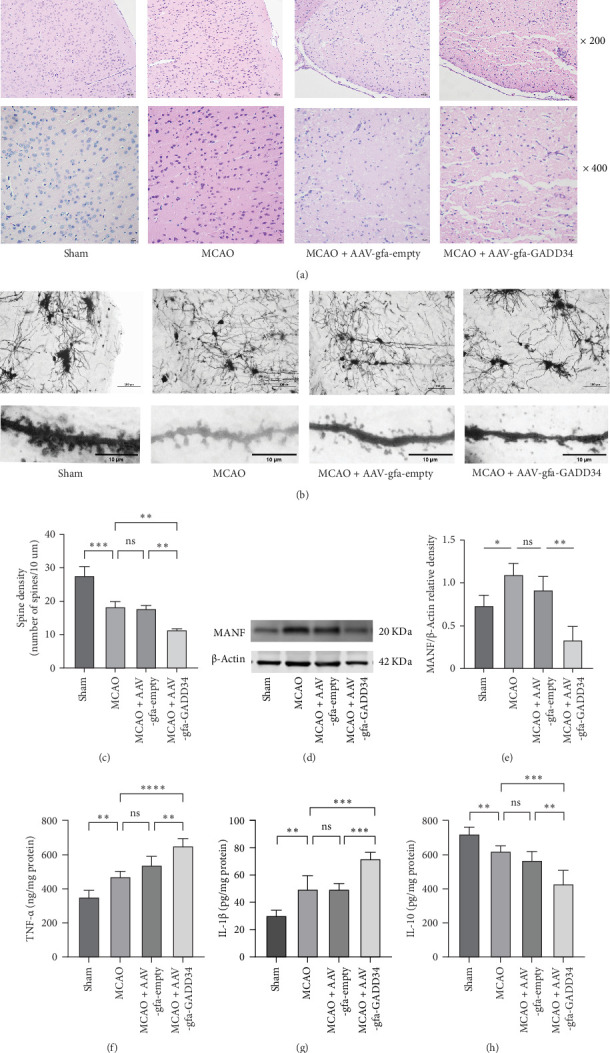
Blockade of PERK signaling pathway in mice astrocytes reduced MANF expression and exacerbated synapse loss and neuronal damage in the peri-infarct zone. (A) Representative images of H&E staining on the 7^th^ day after MCAO. The scale is 100 μm. (B, C) Representative images of Golgi staining and quantitative analysis of dendrite spine density (*n* = 3), and the scale is 100 μm and 10 μm, respectively. (D, E) western blot of representative MANF images and quantitative analysis (*n* = 3). (F–H) ELISA assay was used to detect the expression of inflammatory factors (*n* = 5). ns, Not significant. *⁣*^*∗*^*p* < 0.05, *⁣*^*∗∗*^*p* < 0.01, *⁣*^*∗∗∗*^*p* < 0.001, and *⁣*^*∗∗∗∗*^*p* < 0.0001.

As shown in Figure 2A, H&E staining showed that mice cortical neurons in the Sham group were neatly arranged with intact cell structure and large, regular nuclei. Mice cortical neurons in the MCAO group showed enlarged nuclei and nuclear chromatin condensation, and the number of neurons was reduced. Compared with the MCAO group, the MCAO + AAV-gfa-GADD34 group showed more severe neuronal loss with a large number of vacuoles around the cells. Besides, dendritic spine density affected synaptic plasticity. As depicted in Figure 2B,C, at 7 days after surgery, the Golgi staining showed that the density of dendritic spines was significantly reduced in the MCAO group compared to the Sham group, and appeared to be further decreased in the MCAO + AAV-gfa-GADD34 group compared to the MCAO + AAV-gfa-empty group. As shown in Figure 2D,E, western blot showed that the expression of MANF in the peri-infarct zone was elevated in the MCAO group, while this expression was reversed in the MCAO + AAV-gfa-GADD34 group. ELISA results revealed increased expression of IL-1β and TNF-α and decreased expression of IL-10 in the MCAO group compared to the Sham group. The MCAO + AAV-gfa-GADD34 group had higher levels of pro-inflammatory factors IL-1β and TNF-α and lower levels of anti-inflammatory factor IL-10 compared to the MCAO + AAV-gfa-empty group (Figure 2).

### 3.3. Activation of the PERK Signaling Pathway in Astrocytes Promoted MANF Expression, Upregulated Synaptic Proteins Expression, and Enhanced Cell Viability, While Knockdown of MANF Reversed These Effects

**Figure 3:**
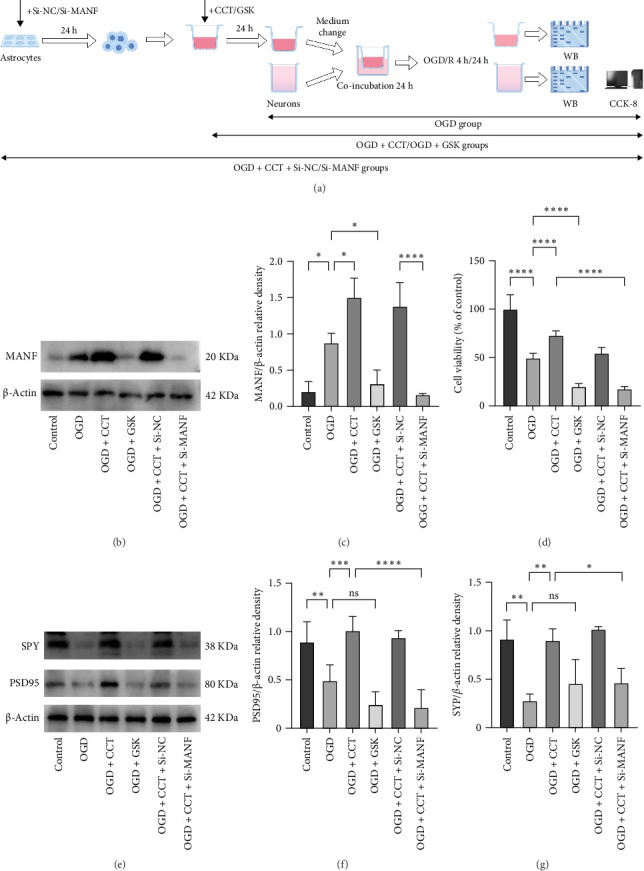
Activation of the PERK signaling pathway in astrocytes promoted MANF expression, upregulated synaptic proteins expression, and enhanced cell viability, while knockdown of MANF reversed these effects. (A) Grouping and experimental procedure. (B, C) Western blot analysis of MANF in astrocytes (*n* = 5). (D) CCK8 detected neuronal viability (*n* = 6). (E–G) Western blot analysis of synaptic proteins PSD95 and SYP in neurons (*n* = 5). ns, Not significant. *⁣*^*∗*^*p* < 0.05, *⁣*^*∗∗*^*p* < 0.01, *⁣*^*∗∗∗*^*p* < 0.001, *⁣*^*∗∗∗∗*^*p* < 0.0001.

An astrocyte-neuron cocultures system was established and exposed to OGD (Figure 3A). As shown in Figure 3B,C, activation of the PERK signaling pathway using CCT020312 increased MANF expression compared to the OGD group, and conversely, GSK2656157 downregulated MANF expression. As shown in Figure 3, compared with the OGD group, the OGD + CCT group promoted the expression of PSD95 and SYP. However, the opposite trend was observed in the OGD + GSK group, which was consistent with the mice results. Subsequently, knockdown of astrocyte MANF significantly decreased the expression of the PSD95 and SYP compared to the OGD + CCT group. These results confirmed that CCT020312 promoted synaptic proteins expressions by upregulating MANF. The CCK8 results revealed that neuronal viability was decreased in the OGD group than in the control group, and cell viability increased significantly after CCT020312 treatment, while the use of GSK led to a further decreased cell viability. Besides, knockdown of MANF resulted in a significant decrease in neuronal cell viability compared to the OGD + CCT group (Figure 3D).

### 3.4. Knockdown of MANF in Astrocytes Increased STAT3 Phosphorylation and Inflammatory Factor Release While Decreasing Neuronal Synaptic Proteins Expression, Whereas rhMANF Supplementation Had the Opposite Effect

**Figure 4:**
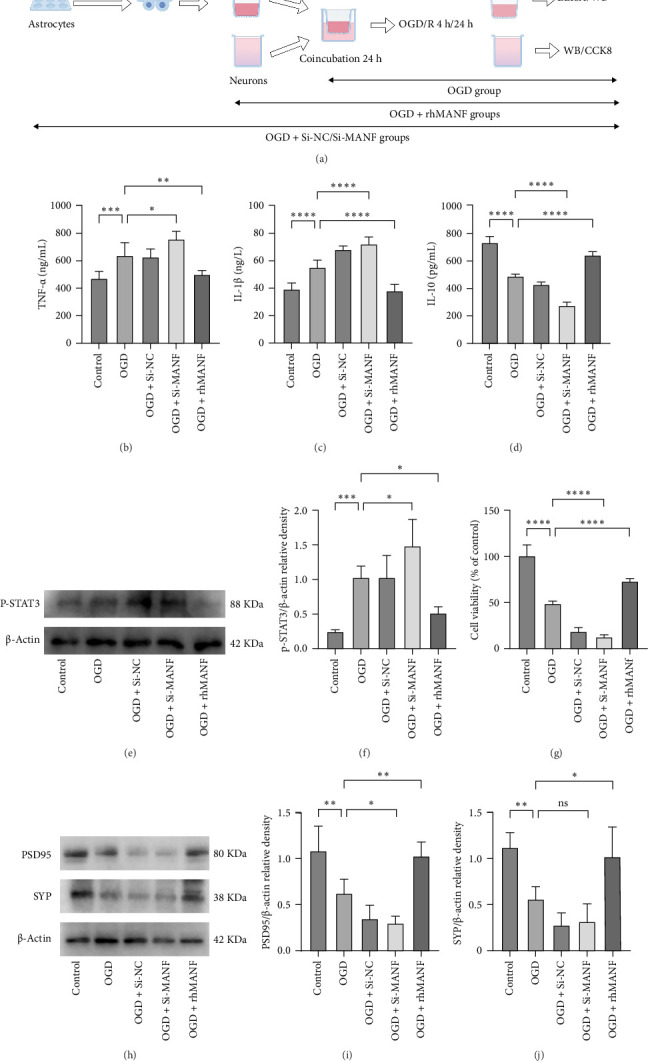
Knockdown of MANF in astrocytes increased STAT3 phosphorylation and inflammatory factors release while decreasing neuronal synaptic proteins expression, whereas rhMANF supplementation had the opposite effect. (A) Grouping and experimental procedure. (B–D) Enzyme linked immunosorbent assay was used to detect the expression of inflammatory factors in astrocytes (*n* = 6). (E, F) Western blot analysis of STAT3 phosphorylation in astrocytes (*n* = 5). (G) CCK8 detected neuronal viability (*n* = 6). (H–J) Western blot analysis of synaptic proteins PSD95 and SYP expression in neurons (*n* = 5). ns, Not significant. *⁣*^*∗*^*p* < 0.05, *⁣*^*∗∗*^*p* < 0.01, *⁣*^*∗∗∗*^*p* < 0.001, *⁣*^*∗∗∗∗*^*p* < 0.0001.

After knockdown of MANF in astrocytes or pretreatment of astrocytes with rhMANF, the culture medium was replaced and the coculture system was established with neurons, and the flowchart is shown in Figure 4A. The OGD group increased pro-inflammatory factors IL-1β and TNF-α while decreasing anti-inflammatory factor IL-10 in astrocytes compared to the control group. Knockdown of MANF showed more severe results than the OGD group, whereas rhMANF supplementation reduced IL-1β and TNF-α levels while increasing IL-10 levels (Figure 4). In addition, as shown in Figure 4E,F, OGD promoted STAT3 phosphorylation in astrocytes compared with the control group. Compared with the OGD group, knockdown of MANF exacerbated STAT3 phosphorylation, while supplementation of rhMANF attenuated STAT3 phosphorylation. The CCK8 results showed that the OGD group reduced the neuronal viability. Compared with the OGD group, knockdown of MANF further reduced cell viabillity, while supplementation of MANF enhanced cell viability (Figure 4G). The expression of PSD95 and SYP was reduced in the OGD group compared to the control group. Compared with the OGD group, PSD95 was further decreased after knockdown of MANF; while rhMANF administration increased the expression of PSD95 and SYP (Figure 4).

## 4. Discussion

Our in vivo and in vitro experiments investigated the role and mechanism of astrocytes in neuronal synaptic remodeling and neurological function recovery in the acute phase after stroke. We found that astrocytes can activate the PERK-elf2α signaling pathway to produce MANF. This inhibited STAT3 phosphorylation, suppressed the release of inflammatory factors, increased neuronal synaptic protein expression, reduced synaptic loss, and promoted neurological function recovery after stroke.

Apart from thrombolytic therapy in the acute phase, treatment options for patients with ischemic stroke are still very limited. This underscores the urgency of enhancing endogenous remedies. Endoplasmic reticulum stress is prevalent in neuronal cells after stroke [27]. Previous studies have focused on the role of neuronal endoplasmic reticulum stress in endogenous repair after stroke [28, 29], whereas the role of endoplasmic reticulum stress in astrocytes after stroke is less well studied and controversial. One study reported that blocking the PERK signaling pathway in astrocytes inhibited the conversion of astrocytes to type A1, improved synaptic function, and increased survival in mice with prion disease [30]. However, another study concluded that astrocytes activate the PERK signaling pathway by upregulating CNβ protein expression and reduce infarct size in a mouse stroke model [31]. In addition, Lahiri et al.'s [32] study confirmed that astrocytic PERK deficiency exacerbated neurological impairment after stroke in aged mice. In our mice MCAO model, we found that blocking the PERK/elf2α signaling pathway in astrocytes exacerbated infarct size, decreased dendritic spine density in neurons, and inhibited the recovery of neurological function, which is consistent with the findings of Lahiri et al. [32]. Meanwhile, we demonstrated that activation of the PERK/elf2α pathway using CCT020312 rescued the decline in synaptic proteins and improved neuronal survival after OGD.

After MCAO, PERK activation levels was increased and synaptic protein expression remained decreased in brain tissue compared with the Sham group, while a further decrease occurred after blocking the astrocytic PERK pathway. We hypothesized that after cerebral infarction, endoplasmic reticulum stress could occur in almost all cells, thereby, activating the UPR-PERK pathway. However, the strength of the benefits and disadvantages exhibited by individual cells remained to be elucidated. Jin et al. [33] found that after cerebral infarction, endoplasmic reticulum stress could occur in glial cells and macrophages in addition to neurons. Following ischemic stroke, a number of pathological processes are activated, including oxidative stress, ER stress, and mitochondrial dysfunction. Many of these processes are regulated by the PERK pathway. Li et al. [34] demonstrated that Hes1 knockdown caused neuronal apoptosis and neurological deficits by promoting activation of the PERK/elF2α/ATF4/CHOP pathway, which was ameliorated by blockade of PERK phosphorylation. PERK has been demonstrated to play a regulatory role in a number of different pathways. The decrease in synaptic proteins and behavioral deficits after cerebral infraction are the result of a combination of these regulatory effects. Further investigation is necessary to elucidate the specific mechanisms of PERK association in different cells.

MANF, also known as arginine-rich mutated in early stage of tumors (ARMET), is a neurotrophic factor regulated by endoplasmic reticulum stress. After stroke, endoplasmic reticulum stress occurs in neurons, astrocytes, and microglia, and MANF expression was found to be upregulated in all of these cells [35]. Many studies had shown that MANF plays a protective role in neuronal regeneration, maturation, and differentiation after stroke. MANF inhibited inflammatory responses and improved blood-brain barrier integrity after stroke in aged mice via the TLR4/MyD88/NF-κB pathway [36]. Another study reported that administration of MANF increased the number of macrophages in the subcortical region and improved behavioral deficits [37]. In our in vivo study, the overall expression of MANF in the peri-infarct zone increased after stroke. Blocking the PERK pathway in astrocytes decreased MANF expression, inhibited neuronal synaptic regeneration, and neurological recovery. In addition, in vitro experiments further demonstrated that activation of astrocytic PERK pathway using CCT020312 promoted neuronal survival, but silencing of MANF reversed this protective effect, inferring that the promotion of neuronal synaptic remodeling by astrocytes through the PERK pathway is dependent on MANF production.

STAT3 can directly or indirectly activate the gene expression of several inflammatory cytokines, such as IL-6, TNF-α, and IL-1β, which are key mediators of the inflammation. STAT3 was found to be upregulated in astrocytes after stroke [38], but the role of STAT3 in astrocytes and its effect on neuronal synaptic remodeling are unclear. Chen et al. [39] demonstrated that running regulated neuronal synaptic remodeling by activating astrocytic STAT3-GPC6 axis. Besides, overexpression of MANF induced neural stem cell differentiation, increased cell migration in SVZ explants, activated the STAT3 signaling pathway, and promoted endogenous repair after stroke [14]. However, some studies presented some different viewpoints, Mina et al. found that activation of STAT3 in astrocytes after stroke promoted synapse loss in the peri-infarct [40]. Shan et al. [41] noted that after OGD, Ex-4 suppressed astrocyte JAK2/STAT3 signaling pathway, which ameliorated ischemia-induced inflammation and disruption of blood-brain barrier. Therefore, the role of STAT3 in astrocytes needs to be further investigated in depth. Our study demonstrated that the phosphorylation level of STAT3 was increased in astrocytes during the acute phase after stroke. When we reversed this effect by upregulating MANF through activation of PERK/p-elf2α, the production of proinflammatory cytokines was reduced, thereby, promoting neuronal synaptic remodeling and neurological function recovery. Similarly, the exogenous supplementation of MANF led to a reduction in astrocyte STAT3 phosphorylation, an enhancement in neuronal viability, and an increase in synaptic proteins expression.

The role of the PERK/p-elf2α signaling pathway in different neuronal cells during the acute and chronic phases after stroke is also controversial. Chou et al. [42] noted that in the acute phase of the TBI model, enhanced phosphorylation of PERK and elf2α in hippocampal neurons provided neuroprotection. However, in the chronic phase (4 weeks post injury), the improvement of cognitive function in mice depended on the inhibition of elf2α phosphorylation [42]. In another study, knockdown of neuronal PERK in mice resulted in larger infarct volumes and more severe behavioral deficits in both the acute and chronic phases after stroke, suggesting that activation of the neuronal PERK signaling pathway exerted a protective effect role in both periods [29]. In astrocytes, the role of the PERK signaling pathway in the acute and chronic phases after brain injury is unclear. Lahiri et al. [32] found that specific knockdown of PERK in astrocytes led to decreased activity and feeding and increased mortality in mice on the third day after stroke. Consistent with the results of this study, we found that blocking the PERK/p-elf2α signaling pathway in astrocytes worsened neuronal synaptic remodeling and neurodegeneration on day 7 after MCAO in mice. Meanwhile, activation of PERK in astrocytes improved neuronal survival in the OGD model. Taken together, our study confirms that activation of the PERK-elf2α signaling pathway in astrocytes during the acute phase after stroke inhibits the release of inflammatory factors by generating MANF and inhibiting STAT3 phosphorylation, thereby, increasing neuronal synaptic proteins expression, reducing synapse loss, and promoting neurological recovery.

Our study also has some shortcomings. First, we have only focused on the role of the PERK pathway in neuronal synaptic remodeling and neurological recovery of astrocytes in the acute phase after stroke, and the role of the PERK pathway on neuronal synaptic remodeling in the chronic phase still needs to be revealed in depth. In addition, in future studies, analysis of the downstream pathways following STAT3 phosphorylation in astrocytes will provide more specific mechanisms to better understand the role played by astrocytes after stroke.

## 5. Conclusion

Our study indicates that after stroke, astrocytes activate PERK and upregulate MANF expression, which inhibits STAT3 phosphorylation, reduces the release of pro-inflammatory cytokines, rescues neuronal synapse loss, and promotes the recovery of neurological function in mice. This provides a new perspective for understanding stroke and rehabilitation, and helps to find endogenous treatment methods to combat stroke and minimize its consequences.

## Data Availability

The data that support the findings of this study are available from the corresponding author upon reasonable request.
